# Emerging roles of PHLPP phosphatases in lung cancer

**DOI:** 10.3389/fonc.2023.1216131

**Published:** 2023-07-27

**Authors:** Xinhang Xia, Wenhu Pi, Meng Chen, Wei Wang, Danyang Cai, Xuequan Wang, Yanli Lan, Haihua Yang

**Affiliations:** Key Laboratory of Radiation Oncology of Taizhou, Radiation Oncology Institute of Enze Medical Health Academy, Department of Radiation Oncology, Taizhou Hospital Affiliated to Wenzhou Medical University, Taizhou, Zhejiang, China

**Keywords:** PHLPP, lung cancer, phosphatase substrate, therapeutic target, potential effect

## Abstract

Pleckstrin homologous domain leucine-rich repeating protein phosphatases (PHLPPs) were originally identified as protein kinase B (Akt) kinase hydrophobic motif specific phosphatases to maintain the cellular homeostasis. With the continuous expansion of PHLPPs research, imbalanced-PHLPPs were mainly found as a tumor suppressor gene of a variety of solid tumors. In this review, we simply described the history and structures of PHLPPs and summarized the recent achievements in emerging roles of PHLPPs in lung cancer by 1) the signaling pathways affected by PHLPPs including Phosphoinositide 3-kinase (PI3K)/AKT, RAS/RAF/mitogen-activated protein kinase (MEK)/extracellular signal-regulated kinase (ERK) and Protein kinase C (PKC) signaling cascades. 2) function of PHLPPs regulatory factor USP46 and miR-190/miR-215, 3) the potential roles of PHLPPs in disease prognosis, Epidermal growth factor receptors (EGFR)- tyrosine kinase inhibitor (TKI) resistance and DNA damage, 4) and the possible function of PHLPPs in radiotherapy, ferroptosis and inflammation response. Therefore, PHLPPs can be considered as either biomarker or prognostic marker for lung cancer treatment.

## 1 Introduction

Protein phosphorylation, which was first discovered in 1955, is one of the most important post-transcriptional modifications. It plays very important roles in regulating cell cycle, growth, proliferation, differentiation and other cell activities (1). Abnormal protein phosphorylation is one of the hallmarks of cancer cells and, in many cases, is a prerequisite for maintaining tumor development and progression. Protein phosphatases are classified into several types, such as serine/threonine (Ser/Thr), tyrosine, and histidine, depending on the primary structure of the enzyme and the specificity of its substrate in different phosphorylated amino acids. pSer and pThr account for more than 98% of all human phosphorylated residues (2). Ser/Thr phosphatases consist of three distinct groups of phosphoprotein phosphatase (PPP), metal dependent protein phosphatase (PPM), and haloacid dehalogenase (HAD) (3, 4).

The pleckstrin homology domain (PH domain) leucine-rich repeat protein phosphatase PHLPP, which was first found in 2005, can specifically dephosphorylate AKT, therefore, PHLPP can regulate AKT signaling to modulate the AKT genes’ activity (5). PHLPP has two isoforms named PHLPP1 and PHLPP2. Over-expression of PHLPPs could result in significant inhibition of cell growth in glioblastoma by dephosphorylating AKT (5). Since then, more discoveries of PHLPPs have been verified to have achievements in the regulation of cell proliferation, triggering apoptosis and suppressing tumor growth and so on in different solid tumors such as prostate cancer, colon cancer and pancreatic cancer (PHLPPs acts as a tumor suppressor gene) (6–9). PHLPP has been considered to be a potential therapeutic indicator for those type of tumors. However, PHLPP has not been fully studied in Lung cancer (LC), which was the leading of morbidity and mortality of cancer in the world (10, 11).

LC mainly includes non-small cell lung cancer (NSCLC), which account for 80% of the total, and small cell lung cancer (SCLC) (12, 13). In NSCLC, lung adenocarcinoma (LUAD) is the most common subtype, accounting for about 60% in China, and lung squamous cell carcinoma (LUSC) is the second most common subtype (13). The occurrence of lung cancer is related to age, smoking, heredity and other factors. EGFR and Kirsten rat sarcoma viral oncogene (KRAS) mutations are the two most common changes found in genetic diagnosis of lung cancer patients (14). Unfortunately, the rate of diagnosis is low in the early stages. Standard therapy includes thoracic radiotherapy, chemotherapy, immunotherapy and TKIs, has been used more for the treatment of LC. Although LC patients had good responses to those treatments but easily develop radio-resistance or drug resistance. Factors, such as gene and inflammation, have been explored to associate with the resistance by changing of signaling pathways in tumor cell DNA repair, antitumor immune response, and the formation of local tumor microenvironment (TME). Over the past decade, PHLPPs has been proved to associate with DNA repair, inflammation and immune response in different solid tumors (15–19). In recent years, researchers (including authors) paid special focus on the PHLPPs in LC. The relationship between PHLPPs and LC has been explored form basic research and clinical study (20, 21). Even the evidence may not very strong, this review is trying to discuss the potential mechanisms of PHLPPs in LC, highlights the emerging findings of PHLPPs as a potential target in radiotherapy, immune response, and inflammatory activation for the LC treatment, expand the scope of its clinical applications and provide direction for further study.

## 2 Structures of PHLPPs

**Figure 1:**
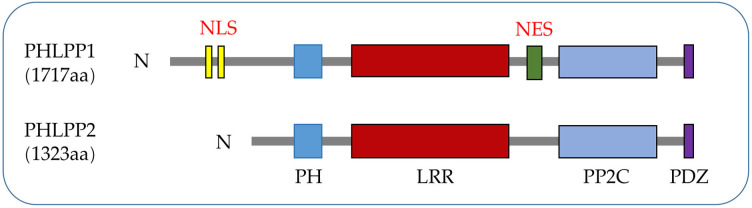
PHLPPs protein structures. Both isozymes have similar domain structures, including a PH domain, a LRR domain, a PP2C phosphatase domain, and a PDZ-binding motif while NLS and NES are specific structures of PHLPP1.

PHLPPs was originally discovered as a direct dephosphorylated protein of AKT, which inhibited cell proliferation through the AKT/PI3K pathway (5). The PHLPPs family include PHLPP1 and PHLPP2 gene subtypes. Both members of the PHLPP family share several similar conserved domains including a PH domain, a hydrophobic leucine-rich repeat (LRR) region, a protein phosphatase 2C (PP2C) domain, and a PSD-95/Disc-large/ZO-1 (PDZ) binding motif (5) while PHLPP1 has N-Terminal Extension (NTE) region. Owing to the relatively low catalytic efficiency of the phosphatase domain (22), the close interaction between the phosphatase domain and the substrate mediated by the regulatory domain is crucial for the action of PHLPPs (23) (**Figure 1**).

### 2.1 NTE domain

The N terminus of PHLPP1 has about 50 kDa of unknown function structure called N-terminal extension (NTE). Either directly binding PHLPP1 or indirectly binding through other scaffold proteins can pass through this domain (24). The function of NTE is to switch the PHLPP1 protein interaction network during mitosis (25). NTE is a substrate for cyclin-dependent kinases 1 (Cdk1) and dissociates from plasma membrane scaffolds such as Scribble (26). When NTE approaches and acquires centromeric and mitotic spindle proteins, it affects cell mitosis and inhibits normal cell division cycles, which is associated with cancer progression and poor prognosis (27–29). Despite being evolutionarily low conserved, NTE is necessary for the recently discovered dephosphorylation of PHLPP1 transcriptional regulatory substrate signal transducer and activator of transcription 1 (STAT1) in colitis (24) (**Figure 1**).

### 2.2 PH domain

PH domain is present in many kinases, phospholipase C, nucleotide exchange factors, GTPases and the GTP-binding proteins which interact with many proteins (30). The PH domain of PHLPPs, a globular structure containing about 120 amino acids, is acquired relatively late in evolution compared to other domains and is found only in mammals (31, 32) (**Figure 1** and section 3.2 for the role of this domain in LC).

### 2.3 LRR domain

**Figure 2:**
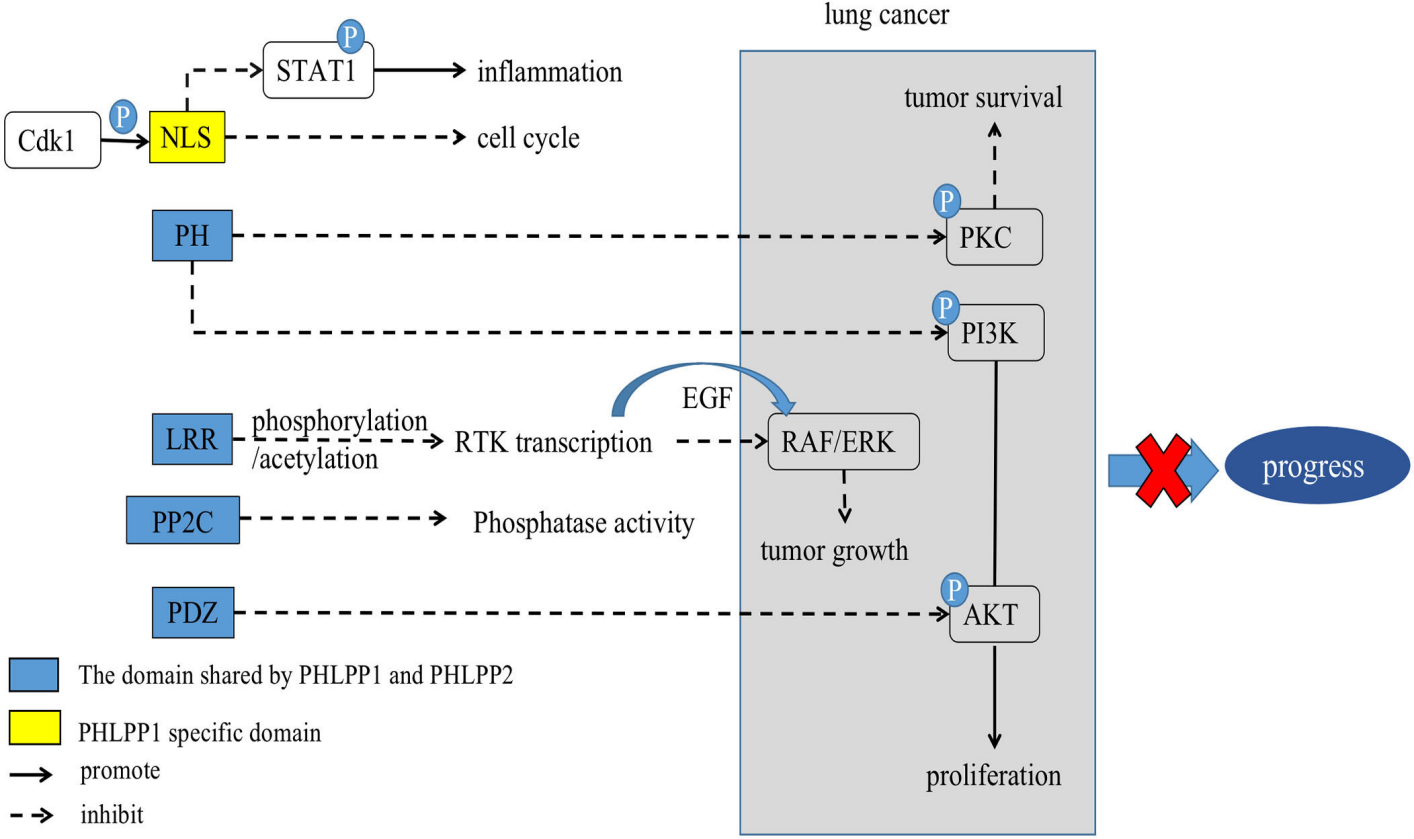
Scheme of corresponding action of PHLPPs and their substrates in lung cancer. The PH domain, LRR domain, PP2C phosphatase domain, and PDZ-binding motif are shared by PHLPPs while NLS and NES are specific structures of PHLPP1.

The LRR domain of PHLPPs isozymes is characterized by multiple repeated leucine residues at the same location. It consists of 18 repeated amino acid sequences (33). This domain regulates transcription of receptor tyrosine kinase (RTK) such as Epidermal growth factor receptor (EGFR) (34) (**Figures 1**, **2**), and interact with KRAS to block the activation of ERK (35) (**Figure 1** and section 3.3).

### 2.4 PP2C domain

The main role of the PP2C domain is to resist the action of common serine/threonine phosphatase inhibitors, resulting in drug resistance (36). Both PHLPP1 and PHLPP2 are members of the PP2C family and are insensitive to most common phosphatase inhibitors (37). Thus, PHLPPs can act as a tumor suppressor proteins as a member of the PP2C family, which is mainly involved in cell growth and stress signaling pathways (38). In PHLPPs, the catalytic effect of PP2C domain alone is low (22), but when it co-exists with the PDZ-binding motif, it become more essential for scaffold binding and AKT dephosphorylation (37) (**Figure 1**).

### 2.5 PDZ domain

In PHLPPs family members, the PDZ ligand is critical for the dephosphorylation of AKT in cells (5). PDZ ligands of PHLPP1 and PHLPP2 bind to scaffold Na+/H+ exchanger Regulatory Factor 1 (NHERF1) (7) due to localization of phosphatases near the membrane, where they could dephosphorylate AKT (39). Both PHLPP1 and PHLPP2 selectively dephosphorylate the same site on AKT despite acting on different AKT subtypes (37, 40). See section 3.1 (**Figure 1**).

## 3 Substrates and regulation of PHLPPs

Different domains of PHLPPs have different functions in the development of tumor, PHLPPs act the carcinogenic or tumor suppressive effect through these structures. At present, it has been found that PHLPPs’ active substrates include AKT, PKC, MAPK/ERK. (5, 24, 31, 32) Those substrates were found to have anti-tumor or pro-tumor effects in lung cancer. Thus, we will discuss the relevant pathways of their actions in LC.

### 3.1 PI3K/AKT

**Figure 3:**
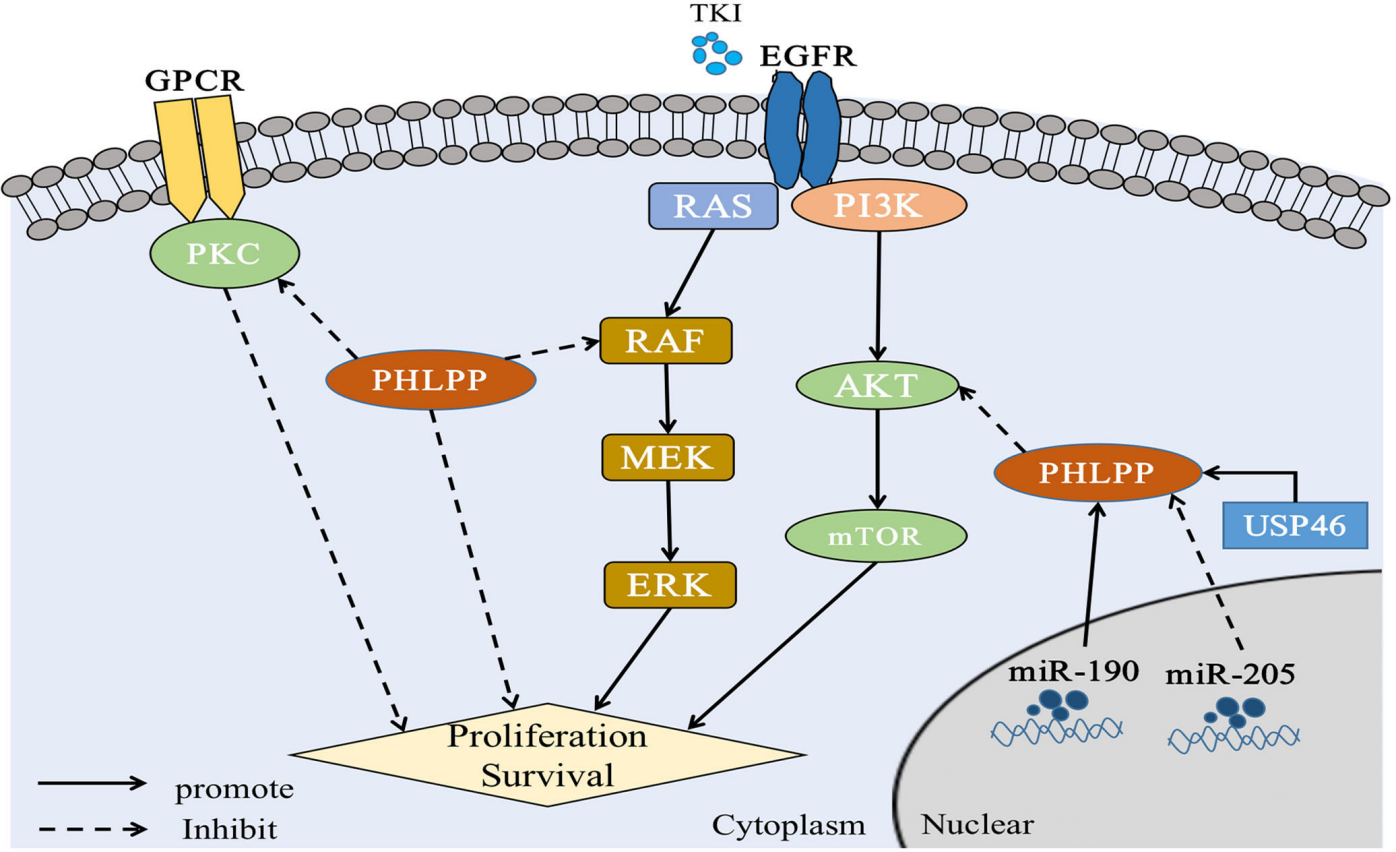
PHLPP substrate and function in lung cancer PHLPP inhibits the proliferation and survival of NSCLC cells by inhibiting RAS/RAF/ERK and AKT, while PHLPP inhibits PKC to resist the anti-tumor effect of PKC. At the same time, USP46 and intracellular miR-190 can induce the expression of PHLPP and thus inhibit the activity of AKT phosphatase to play an antitumor role. On the contrary, intracellular miR-205 can inhibit the expression of PHLPP and act as an oncogene. In addition, for tumor cells, radiation therapy and immune checkpoint therapy may cause cell ferroptosis in a manner that remains to be demonstrated.

PI3K contributes to a variety of cellular processes including cell growth, migration, differentiation, and proliferation by recruiting proteins with PH domains to the plasma membrane (41) (**Figure 2**). One of the most critical and versatile protein kinases in higher eukaryotes is protein kinase AKT (42). Many of AKT’s downstream related substrates have been shown to be involved in cell metabolism, cell survival, cell proliferation and migration (43). Stimulating activation of the PI3K/AKT/mTOR pathway is a key event in the development of cancer especially lung cancer (44–46). Thus, it causes cancer insensitive to treatment (**Figure 3**).

Based on the past research, PHLPPs mainly produce dephosphorylated AKT through the PDZ domain (5). The AKT isomer was investigated and found that PHLPP1 selectively dephosphorylated AKT2, whereas PHLPP2 selectively dephosphorylated AKT1 (47). The ultimate dephosphorylation outcome can inhibit tumor cell growth, thereby prolonging patient survival (48) (The specific role of PHLPPs in LC through this pathway was described in section 4).

### 3.2 PKC

PKC is widely involved in cell survival, mitosis, motility, and invasion. This kinase was initially described as an oncogene and subsequently identified as a cancer biomarker, showing overexpression of PKC in multiple epithelial tumors, including lung cancer (49). Abnormal expression of PKC contributes to the initiation and progression of cancer (50, 51). By database analysis, PKC expression was associated with poor prognosis in KRAS-mutated LUAD (52). PKC is also a highly specific oncogene, it is a key factor in the sensitivity of the body to chemical carcinogens and the induction of carcinogenesis by gene mutation. In the absence of PKC, the number of KRAS-driven and chemical carcinogen-induced lung diseases was reduced(52). More, PKC is essential for the normal physiological response of many cells in lung including permeability, migration, proliferation, apoptosis, and secretion (53, 54).

As showed the PKC function above, we consulted the relationship between PKC and PHLPP. Both PHLPP1 and PHLPP2 can dephosphorylate the hydrophobic motif of PKC, leading unstable and degradation of PKC protein (31, 55). Loss of PHLPPs phosphatase function could lead to the increasing of PKC protein level, thus promoting tumorigenesis (31, 56). Depletion of PHLPPs in cells leaded to the AKT phosphorylation and increased total PKC levels (55, 57). A study showed that PKCα was fully phosphorylated at the PHLPP site, and PHLPP1 levels were negatively correlated with PKC in more than 5,000 tumor patients (58). Other studies also showed that PHLPP affected disease progression through the PKC pathway (59). Those findings provided evidence that PHLPP was a potential tumor suppressor (**Figures 2**, **3**).

### 3.3 MAPK/ERK

During disease progression, upstream RTK signals such as EGF stimulate tyrosine kinase receptors, which in turn cause activation of RAS, and further activates RAF/MEK/ERK proteins through kinase cascades (60). The MAP kinase (MAPK)/ERK pathway has been shown to play an important role in the growth and differentiation of tumor cells and to play an effective carcinogenic role in LUAD (61–63). In colorectal cancer, one study reported that both PHLPP1 and PhLPP2 dephosphorylated RAF1, thereby inhibiting colorectal cancer proliferation. When PHLPP1 and 2 were knocked out, the expression of epithelial-mesenchymal transition (EMT) markers was increased, which promoted the migration and invasion of colorectal cancer, tumor proliferation and progression and meanwhile negatively regulate MEK signaling pathways (64).

Simultaneous mutations of v-Raf murine sarcoma viral oncogene homolog B (BRAF) and KRAS, two key oncogenes in RAS/RAF/MEK/ERK signaling pathway, had been found in NSCLC (65, 66). Previous studies have reported that PHLPP1 could directly interact with KRAS through its LRR domain to negatively regulate ERK pathway (35, 67). PHLPPs also reduces ERK phosphorylation. Our previous patient studies have shown that highly expressed PHLPP1 can have a longer duration of EGFR- TKI resistance, which mainly through MAPK/ERK signaling pathway. It was found that PHLPP1 had a negative correlation with phosphorylated ERK expression in LUAD patients. Similarly, *in vitro* studies, it was found that overexpression of PHLPP1 inhibited EGFR-TKI resistance in non-small cell lung cancer *via* the MAPK/ERK pathway (20, 21). See section 4 (**Figure 3**).

However, the current research is still limited. Since the discovery of PHLPPs, a lot of studies have been conducted on illuminating of its substrates, including STAT1, MST1, S6K1 and so on (24, 68, 69). Although these substrates were associated with the development of lung cancer (70–72), there was no direct evidence that they were associated with PHLPPs in LC. In addition, in a global PHLPP knocked out (PHLPP^-/-^) mouse model, researcher has focused more on the gastrointestinal tract, including the progress of cancer and the changes of immune cells in the immune microenvironment.

## 4 Therapeutic applications and prognostic prediction of PHLPPs in lung cancer

PHLPPs was originally discovered as a phosphatase that regulated the phosphorylation of hydrophobic proteins, thereby terminated the PI3K-AKT signaling pathway (5). While mis-regulation of AKT pathway was an important factor in tumorigenesis (73, 74). Both PHLPPs genes are located on chromosomal regions which commonly lost in different type of cancers, indicating PHLPPs have tumor suppressor function potential (75). Here we concluded the related targets of PHLPPs in lung cancer based on existing studies.

At first, PHLPPs was discovered as a direct dephosphorylated protein of AKT (5). According to this target, relevant clinical and basic research has been carried out to prove that PHLPPs can be used as a potential therapeutic target and a prognostic prediction marker to participate in treatment methods including drug therapy and radiotherapy (75) (**Table 1**).

**Table 1:** Progress and related studies of PHLPP in NSLCL.

**Function** **study**	**Sample**	**Subtypes**	**Association with lung cancer**	**Target point pathway**	**Reference**
clinical sample	LUAD	PHLPP1	High PHLPP1 is positively correlation with good prognosis	N/A	(76)
clinical sample	resistance to EGFR-TKIs in LUAD	PHLPP1	High PHLPP1 Predicted higher survival and longer duration of acquired resistance	N/A	(20)
clinical sample and TCGA	LUAD	PHLPP2	Low PHLPP2 expression is associated with disease progression and metastasis	N/A	(77)
basic rese-arch	resistance to EGFR-TKIs in LUAD cell line	PHLPP1	Down-regulated PHLPP1 can prolong the acquired resistance time of LC cells to EGFR-TKI through PI3K-AKT and MAPK-ERK pathways	PI3K-AKT and MAPK-ERK	(21)
basic rese-arch	LUAD and LUSC cell line	PHLPP1	Radiation inhibits cell proliferation through the USP46-PHLPP1-AKT pathway	USP46-AKT	(19)
basic research	Transcriptional miR-205 in LUAD and LUSC cell line	PHLPP2	PHLPP2 is inhibited by upregulated miR-205 and promotes cell proliferation through activation of AKT/FOXO3a and AKT/mTOR signaling pathways	AKT/FOXO3a and AKT/mTOR	(78)
basic research	Transcriptional miR-190 in lung epithelial cell line	PHLPP1	miR-190 lead to the downregulation of PHLPP1, which leads to the enhancement of the Akt regulatory protein vascular endothelial growth factor (VEGF), promoting tumor progression	AKT	(79)

### 4.1 Clinical research

Since its discovery, PHLPPs has been proved to play a role in a variety of solid tumors, including colorectal (6, 64, 80), prostate (7, 81, 82), breast (83), and so on. Among lung cancers, NSCLC accounts for a larger proportion, with a higher incidence of LUAD, accompanied by the most common EGFR mutation. Based on the above basic research effect of PHLPPs, we summarize its clinical application in LUAD.

#### 4.1.1 LUAD with PHLPP1

LUAD is the most common type of lung cancer. Based on an experiment performed before, in which 158 specimens from patients with LUAD collected from 2008 to 2010 in a single clinical center, and the specimens were stained with immunohistochemical markers of PHLPP1, p-AKT and p-ERK, increased PHLPP1 expression was found and it was associate with decreased invasiveness of tumor and better survival of LUAD patients (76). The mean survival time and 3-year survival rate of patients with high or low PHLPP1 expression were compared, and it was found that patients with high expression were significantly higher than patients with low expression (45 month versus 38 month)(76). (**Table 1**) Mechanismly, those effect might be due to the PHLPP1 expression was negatively correlated with p-AKT or p-ERK, which was involved in tumor progression and were associated with aggressiveness in advanced lung cancer.

#### 4.1.2 EGFR-TKIs resistance with PHLPP1

Activation of EGFR signaling pathway was associated with tumor growth and proliferation (84). EGFR-activating mutations was most common in patients with lung cancer, especially LUAD. TKI produced based on this mutation like gefitinib, erlotinib and afatinib has better objective response rate than conventional chemotherapy (85). Since there are no early diagnostic indicators for NSCLC, most patients are found in advanced stage without surgery potential, therefore, EGFR-TKIs were used for those patients with beneficial effect but some patients still had a poor prognosis rate (86–88). This might be due to the development of drug resistance (89, 90), and the reason was because of the MAPK/ERK signaling pathway was activated by EGFR and this activation stimulates PI3K/Akt activation (91).

Previous studies have demonstrated that PHLPP1/2 was capable of dephosphorylating RAF in colorectal cancer and increasing tumor invasion and migration by negatively regulating RAS/RAF/MEK/ERK signaling pathways (5, 64). In a previously our patient sample study, an analysis of 75 patients with advanced LUAD who received EGFR-TKIs, high PHLPP1 expression have better Progression-Free-Survival (PFS) and Overall survival (OS) than those whose PHLPP1 expression was low. Those results suggest that the survival time was better, and the acquired resistance time was longer in patients with high PHLPP1 expression in EGFR TKIs targeted therapy than low expression(20) (**Figure 3** and **Table 1**). Meanwhile the PI3K/AKT pathway protein was also detected, and we found the same results as before in the LUAD patients (see section 4.1.1).

#### 4.1.3 LUAD with PHLPP2

In recent years, many studies have shown that PHLPP2 is generally not expressed in a wide-ranging of cancer biological processes such as cancer cell proliferation, cancer metastasis and tumorigenesis (92, 93). One study showed that the expression of PHLPP2 was decreased in NSCLC tissue, and it was significantly lower than in normal lung tissue. That is inconsistent with the phenomenon observed in The Cancer Genome Atlas (TCGA) data base. Meanwhile, the result showed that low PHLPP2 expression was one of the key factor leading to poor OS in NSCLC patients, the pathological staging (pTNM) staging and PHLPP2 expression were independent prognostic factors for OS (77). Low PHLPP2 protein level predicted poor survival of NSCLC and a poor prognosis in LUAD (77). In conclusion, study demonstrated that high levels of PHLPP2 expression can predict better survival outcomes and is expected to become a new screening indicator for NSCLC (**Figure 3** and **Table 1**).

### 4.2 Basic research

#### 4.2.1 Drug resistance

LUAD accounts for a large proportion of lung cancers. Nearly half of LUAD have EGFR mutations (94). EGFR-TKIs are therefore widely used in patients with LUAD (95, 96). Although TKI is more accurate and effective than traditional chemotherapy agents, overall survival after treatment is still poor due to patients’ susceptibility to TKI resistance (21).

In terms of resistance, our previous studies strongly demonstrated that loss of PHLPP1 function was a key factor in TKI resistance. Initial clinical finding indicated that patients with high PHLPP1 expression took longer time to develop resistance (20). Based on this finding, we constructed a gefitinib resistant lung cancer cell line *in vitro* experiment, and we found that PHLPP1 expression level decreased. In contrast, the expression levels of PHLPP1 in drug-sensitive non-small cell lines were significantly higher than that in the drug-resistant group, and EGFR-TKI induced cell death was reduced after PHLPP1 expression was knocked down in those cell lines. Overexpression of PHLPP1 in gefitinib-resistant cell line reversely restored the sensitivity to the EGFR-TKI. Downstream signaling pathway analysis showed that two pathways of PI3K-AKT and MAPK-ERK were activated after overexpression of PHLPP1, and those activation achieved EGFR-TKI resistance both *in vitro* and *in vivo* (21). Interestingly, result of PHLPP1 levels from clinical specimens examination showed that the PHLPP1 loss was associated with poor prognosis and the development of EGFR inhibitor resistance (21) (**Table 1**).

#### 4.2.2 DNA damage

Post-translational modification of proteins plays an important role in biological processes such as cell cycle, DNA damage and repair, and apoptosis (97). Among them, DNA is easily damaged by abnormal factors in the environment or various pathogens (98). After DNA damage occurs, the body will initiate the damage repair process at the same time, but when the damage exceeds the repair capacity, the cell death pathway including apoptosis will be triggered (99). Although DNA damage can lead to genomic instability and eventually lead to disease, on the other hand, it also offers a therapeutic possibility (100, 101). AKT kinase plays an important role in DNA damage and resists the effects of radiation therapy by promoting damage repair (102–104). Ubiquitin-specific protease 46 (USP46), a member of the cysteine protease family with deubiquitinating function, has previously been reported to be associated with neurological disorders (105). Previous research has shown that USP46 antagonized AKT activity by deubiquitinating PHLPP1 in colorectal cancer (15).

On this basis, the expression of both USP46 and PHLPP1 were found in low level in lung cancer. In previously study, both USP46 and PHLPP1 were positively correlated (19). After overexpression of USP46 in lung cancer cell lines, PHLPP1 levels were increased through USP46 deubiquitylation, accompanied by a significant decreasing of AKT phosphorylation and inhibition of cell proliferation(19). More, cell proliferation of USP46 overexpressed cells was also significantly reduced after exposure to ionizing radiation. Level of AKT kinase phosphorylation and the DNA damage marker, gamma-H2AX, were also reduced in USP46 overexpressed cells than that USP46 knockdown cells after ionizing radiation. In general, USP46 can inhibits AKT activity by deubiquitinating PHLPP1, inhibits cell proliferation and DNA damage, thus, it increases the radiation sensitivity (19) (**Figure 3** and **Table 1**).

#### 4.2.3 Transcriptional regulation

MicroRNAs (miRNAs) are small non-coding RNAs that bind to complementary base pairs on messenger RNA (mRNA) to inhibit translation and promote degradation, thus, altering gene expression after transcription (106). Numerous evidence show that miRNAs play a key role in malignant transformation and carcinogenesis of cells(107–109). It has been summarized in detail of the different targets with different miRNAs in lung cancer and their cancer-promoting or cancer-suppressing effects by other reviews (110).

MicroRNA(miR)-205 has previously been found to be highly expressed in LC patients (111), most of whom have LUSC (112). Also, it has been reported that miRNAs associated with cell biological processes. Overexpression of miR-205 in NSCLC cells accelerated tumor cell proliferation and promoted blood vessel formation *in vitro* and *in vivo* (111–113). The expressions of PTEN and PHLPP2 were inhibited after miR-205 was overexpressed in lung cancer cell lines. Cloning and tube formation experiments *in vitro* indicated that cell proliferation and angiogenesis were stronger than those in the control group after miR-205 was overexpressed, and this effect disappeared after miR-205 silencing. The same results were seen *in vivo* (78). This study illustrated while increased miR-205 could directly repress PHLPP2 expression and activate the AKT/Mechanistic Target of Rapamycin (mTOR) signaling pathways in multiple NSCLC cell lines (78) (**Figure 3** and **Table 1**).

Dysregulation of miR-190 is involved in the initiation and progression of cancer, and plays a carcinogenic or anticancer role in a variety of tumors (114). The stable expression of miR-190 in hepatoma cell lines can promote cell proliferation, metastasis, and invasion through PHLPP1, which has also been confirmed *in vivo*(115). It also reported that elevated levels of miR-190 could downregulate PHLPP1 translation through direct interaction of miR-190 with the 3′-UTR region of PHLPP1 mRNA, which resulted in decreased PHLPP1 protein expression and enhanced AKT activation in lung cancer (79) (**Figure 3** and **Table 1**).

## 5 Other potential functions in lung cancer with PHLPP

### 5.1 Radiation

Radiotherapy is the most effective cytotoxic therapy for local solid carcinoma (116–118). Radiation therapy is the primary treatment for patients with inoperable NSCLC and has a better therapeutic prospect than surgery (119). Based on these good clinical phenomena, more and more people have conducted new research on the mechanism under radiotherapy, trying to find more accurate tumor treatment strategies. Radiation therapy can inhibit tumor growth infiltration by affecting the growth cycle, proliferation of tumor cells and tumor angiogenesis, DNA damage or immune activation and other related pathways (120–125). However, radiation resistance during treatment limits the effectiveness of radiation therapy in patients, one of the possible causes of radio-resistance is the abnormal activation pathways in tumor cells during radiotherapy, such as the RTKs-related protein PI3K/AKT (126) which was shown that abnormal AKT activation can promote DNA damage repair and enhance cell resistance (127).

It is well known that the direct target of PHLPPs is AKT, which inhibits tumor proliferation and invasion through dephosphorylation of AKT. Previous study demonstrated that radiation could affect USP46 expression levels and thus altered the expression of PHLPP1 and AKT (19). However, the role of PHLPPs in radiation therapy has not yet been explored. Targeted inhibition of phosphatase activity may be another option to enhance tumor radiosensitivity (128). We speculate that PHLPPs is involved in radiation-induced DNA damage and thus affects tumor proliferation. And that’s the goal we’re going to explore.

**Figure 4:**
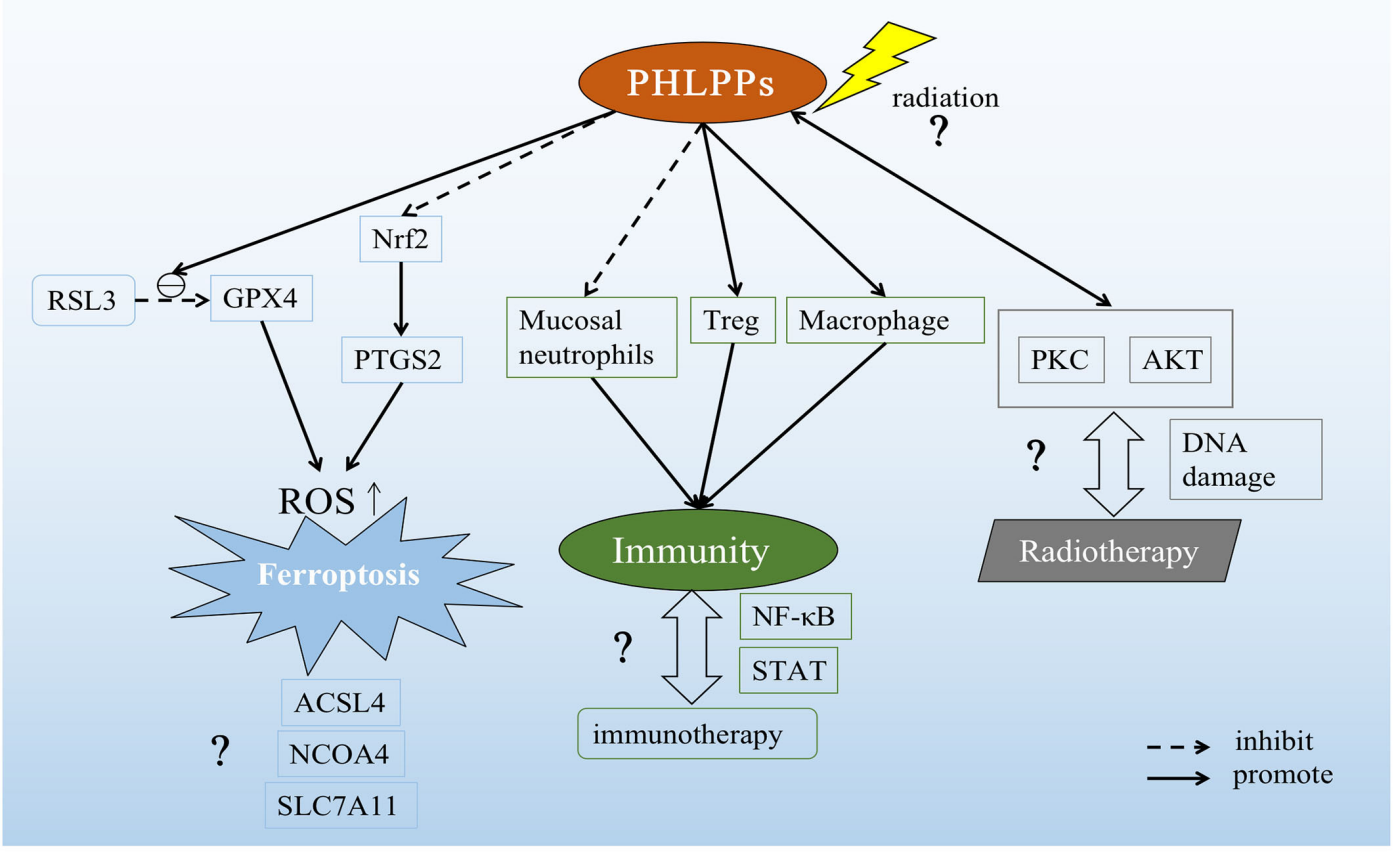
Possible role of PHLPPs as a potential target in lung cancer. PHLPPs can promote the inhibition of RSL3 on GPX4 and inhibit the expression of Nrf2 to increase prostaglandin-endoperoxide synthase 2 (PTGS2). These two effects ultimately lead to the accumulation of ROS and the induction of ferroptosis. But other pathways related to ferroptosis have not been explored. In the immune response pathway, PHLPPs inhibit neutrophils and promote the action of Tregs and macrophages. However, the correlation between PHLPPs and immunotherapy has not been clearly demonstrated in the immune-related pathways NF-κB and STAT that have been explored. On the other hand, the direct effect of radiation on PHLPPs and its effects on PKC and AKT downstream of PHLPPs are not yet known, and the relationship between PHLPPs and DNA damage during radiation therapy is also unclear.

In addition, radiation therapy also caused changes in the PKC pathway. He et al. had demonstrated that ionizing radiation can induce classical PKC (cPKC) activation, thereby promoting RAS/ERK activation (129) and finally apoptosis was promoted and apoptosis-like related markers were changed (130). We have previously discussed the relationship between PHLPP and PKC (see section 3.2). On this basis, the hypothesis that whether radiotherapy can directly act on PHLPPs and induce apoptosis has proposed a new direction for us to explore the effect of PHLPPs in lung cancer (**Figure 4**).

### 5.2 Immune response

In addition, immunotherapy has also been a great success. There have been great advances in treating cancer through immunotherapy over the past decade. The effective way to achieve anti-tumor immunity is to block the bridge between cancer cells and immune cells to activate the body’s immune response. Existing immune checkpoint inhibitors can restore the killing effect of immune cells on tumor cells, especially in lung cancer has entered clinical use (131). However, there are different immune cells in the tumor microenvironment. How to reverse the immunosuppressive cells in the tumor microenvironment to the activated state to kill tumor cells has also become the direction of future research.

Macrophages, as the primary response cells to innate immune response, can use pattern recognition receptors (PRRs) to accurately recognize foreign pathogens and initiate inflammatory responses (132). Therefore, macrophages play an important role in maintaining tissue homeostasis after tissue injury. In a previous study, mice with whole-body knockouts of PHLPP1 showed strong resistance to the lipopolysaccharide (LPS), a component of Gram-negative bacteria, and significantly increased survival time compared with the control group mice. The main reason was that macrophages with PHLPP1 knockout were able to dephosphorylate signal transducers and activators of transcription 1 (STAT1) after being stimulated by LPS, thus increasing the inflammatory response(24). In addition, another mechanism by which LPS enhanced macrophage inflammatory response has been reported due to the regulation of PHLPP1 expression by specificity protein 1 (SP1)(133).

In colitis, the absence of PHLPPs protects the mice from inflammation (16). An in-depth study showed that the mucosal immunity was improved, the migration function of mucosal neutrophils was enhanced in a systemic PHLPPs knockout mouse model. In the same model, PHLPPs was helpful to maintain neutrophilic homeostasis and alleviate colitis reaction (134). In CD4^+^T cells, inhibition of PHLPP1/2 expression can reduce the induction of Treg cells (18).

In NSCLC, a variety of immune cells in the tumor microenvironment participate in the treatment of immune checkpoint inhibitors (ICIs)(135). Although, as previously described, PHLPPs can act as an immune activator or suppressor in immune cells (23), the role of PHLPPs in immunotherapy in lung cancer remains unanswered. This is also a potential mechanism for the future treatment of lung cancer by PHLPPs as a new target (**Figure 4**).

### 5.3 Others

There are many causes of lung cancer, including genetic factors, abnormal gene activation, harmful chemicals in the environment, smoking and so on(136), among which smoking is the main cause of lung cancer. And about 85 percent of lung cancer cases are caused by smoking (137). There was already evidence that particles released after smoking activate nuclear factor-kappaB (NF-κB) *via* myristoylated alanine-rich C kinase substrate (MARCKS) protein, which promotes tumorigenesis and proliferation and survival of lung cancer cells (138, 139). NF-κB was an important regulator of inflammatory processes and also plays a role in lung cancer(140). A previous study showed that deletion of PHLPP2 led to an increase in p-NF-κB p65 in prostate cancer with a PTEN mutation(81). This provided support for our hypothesis that PHLPPs might influence the progression of lung cancer through the NF-κB inflammatory signaling pathway (**Figure 4**).

Cell death forms include apoptosis, necrosis, pyroptosis, autophagy and so on (141–144). As we know, one of the reasons why tumors proliferation and progress is because they inhibit normal cell death. So how to induce different cell death patterns is also an idea of tumor therapy. The newly discovered iron-related cell death named ferroptosis is a new way of cell death (145), which related to the accumulation of reactive oxygen species(ROS) in cytoplasm, characterized by iron accumulation and lipid peroxidation. Ferroptotic cell death morphologically presents a smaller mitochondria but with increased mitochondrial membrane density and reduced or non-existent mitochondria crista, and outer mitochondrial membrane rupture than normal cell (146). In lung diseases including acute lung injury, chronic obstructive pulmonary disease, pulmonary fibrosis, radiation-induced lung injury, asthma, ferroptosis has shown to play a crucial role, and that provide a new treatment ideas(147). Our recent study showed that PHLPP2 regulated ferroptosis through nuclear factor erythroid 2-related factor 2 (Nrf2) pathway to affect apoptosis in LUSC. In addition, PHLPP2 can work with RSL3, a glutathione peroxidase 4 (GPX4) inhibitor, to enhance the inhibition of GPX4 activity, thereby increasing intracellular ROS accumulation and inducing Ferroptosis. However, the interaction between PHLPP2 and ferroptosis has not been explored, including nuclear receptor coactivator 4 (NCOA4), a key protein in iron metabolism pathway, and GPX4/acyl-CoA synthetase long-chain family member 4 (ACSL4), key proteins in Glutathione (GSH) metabolism pathway, and solute carrier family 7 (SLC7A11), a key protein in ROS metabolism pathway(148). The specific role of PHLPPs in inducing ferroptosis in lung cancer cells may provide a new direction for radiotherapy resistance patients in the future, which may be a new site of action for future research (**Figure 4**).

## 6 Summary

In conclusion, PHLPPs can be used as a potential prognostic marker to confirm a good prognosis. Many studies have confirmed that PHLPPs plays an effective role in inhibiting tumor growth and development and cell apoptosis. Over the past few years, this relatively late group of phosphatases has revealed many new targets and functional mechanisms in different pathologies including cancer cell death and changes in immune microenvironment.

In this review, we mainly describe the history and structure of two subtypes of PHLPPs, downstream targeting pathways in NSCLC, including PKC, RAS/RAF/MERK/ERK, and PI3K/AKT pathways. After that, clinical specimens and basic experiments are summarized, and the relationship between PHLPP1/2 and LUAD as well as the expression of EGFR-TKIs resistance are found through the analysis of clinical patient specimens. In terms of basic experiments, we conducted experiments on EGFR-TKIs resistance and the pathway of action of PHLPPs. At the same time, we elucidated related proteins that directly act on PHLPPs in lung cancer, including USP46 through the effect of PHLPP1 on AKT, and the effect of miR-205 on PHLPP2 and miR-190 on PHLPP1. Finally, we conducted a preliminary exploration of the role of PHLPPs in other aspects, including the possible function of PHLPPs phosphatase on radiation resistance in radiotherapy. In terms of immune response around the tumor microenvironment, previous studies have demonstrated the anti-inflammatory role of PHLPPs in immune cells, including macrophages, neutrophils, and Treg cells. At the same time, among ferroptosis discovered in recent years, our preliminary exploration also found that PHLPP2 could regulate ferroptosis through Nrf2 pathway and affect the inhibitory effect of RSL3 on GPX4. In addition, studies have shown that radiotherapy can induced tumor cells to produce ROS and thus induce ferroptosis(145).

Although PHLPP2 has been proved to play a role as an immune regulator, there were no exploratory articles on LC. In addition, only one research article in radiotherapy had demonstrated the indirect role of PHLPP1 in LC, and there was no clear evidence for the role of PHLPP2, and it had not been explored in other diseases except LC. In addition, as an emerging cell death mode of Ferroptosis, only a few results had verified the relationship between PHLPP2 and Ferroptosis in LC, and other aspects were still to be explored. In terms of inflammation, according to our summary and current research results, we have only observed the relationship between PHLPP2 and inflammation-related pathways in other cancers, and there is no relevant evidence in LC, and the immune-related lung damage caused by smoking is relatively extensive, so this aspect can also be explored as a future research direction.

According to our review, the role of PHLPPs in lung cancer has only been preliminarily explored, and other potential mechanisms and immune-related effects have not been specifically explored. And that’s the goal we and others are going to explore.

## Author contributions

XX, WP, and HY contributed to conception and design of the study. XX wrote the first draft of the manuscript. WP edited and modified manuscripts. HY designed the direction of manuscripts. All authors contributed to the article and approved the submitted version.
